# A Quality Improvement Project on Evaluating the Effectiveness of Using Video Triage Calls to Screen New Referrals Received by the Oxleas Perinatal Mental Health Service

**DOI:** 10.1192/bjo.2025.10454

**Published:** 2025-06-20

**Authors:** Maureen Cheruiyot, Alexander Valnarov-Boulter, Afraa Delvi

**Affiliations:** 1 Lewisham and Greenwich NHS Trust, London, United Kingdom; 2 Oxleas NHS Foundation Trust, London, United Kingdom

## Abstract

**Aims:** There is currently no standardised triage process utilised across perinatal mental health services. Oxleas Perinatal Service (located in South East London, offering service across three boroughs) introduced 15-minute video triage calls, where the staff on duty is allocated six calls a day to screen new referrals received by the service. This was introduced to attempt to standardise the triage process, increase service numbers, and improve patient experience. This study aimed to evaluate the effectiveness of this new triage system.

**Methods:** We retrospectively looked at all referrals received between 15/01/2024 to 15/04/2024. 3 standardised feedback questionnaires designed for staff, admin and patients were sent out through SmartSurvey to: 14 staff members on the duty rota, 3 admin staff involved in triaging referrals and 170 patients who attended the video triage calls across the three boroughs. 7 out of 14 staff members, all 3 of the admin staff and 12 patients responded to the SmartSurvey. 105 patients were subsequently contacted by telephone to try and obtain more responses. Following this, an additional 29 patients responded with a total of 41 patient responses obtained.

**Results:** Feedback analysis was grouped into 3 themes: emerging themes from patient, staff and admin responses. Majority of patients reported they preferred video appointments over telephone and felt that the next steps in their care were made clear to them. Patients who were subsequently offered appointments with clinicians expressed how useful the video triage process was. Recurring challenges reported by patients included: poor connectivity, pace of the session and personal preference between video and telephone.

Staff feedback highlighted an enhanced patient experience and information gathering. In addition to this, an increase in service access numbers was also cited. Common barriers reported included: workload pressures, assessments felt repetitive and logistical issues such as wi-fi connectivity.

Overarching themes from admin responses cited a friendly approach for patients and an increase in access numbers. One major challenge highlighted was the workload.

**Conclusion:** Most patients found video triage useful, thereby highlighting a potential for an adapted process to be used across other perinatal services. To address workload pressures, a change has since been introduced where daily triage calls have been reduced from 6 to 4 calls a day. Anecdotal feedback received says that this is much more manageable. We are in the process of measuring the impact of this change, and findings of this will be reported in the near future.

